# Extracellular Vesicle-Derived MicroRNAs’ Value in Diagnosing and Predicting Clinical Outcomes in Patients with COVID-19 and Bacterial Sepsis

**DOI:** 10.3390/ijms27031334

**Published:** 2026-01-29

**Authors:** Martina Schiavello, Barbara Vizio, Ornella Bosco, Chiara Dini, Barbara Gennaro, Anna Trost, Elisabetta Greco, Salvatore Andrea Randazzo, Emanuele Pivetta, Giulio Mengozzi, Giuseppe Montrucchio, Fulvio Morello, Enrico Lupia

**Affiliations:** 1 Department of Medical Sciences, University of Turin, 10126 Turin, Italy; martina.schiavello@unito.it (M.S.); barbara.vizio@unito.it (B.V.); ornella.bosco@unito.it (O.B.); chiara.dini@unito.it (C.D.); barbara.gennaro@edu.unito.it (B.G.); anna.trost@unito.it (A.T.); emanuele.pivetta@unito.it (E.P.); giulio.mengozzi@unito.it (G.M.); giuseppe.montrucchio@unito.it (G.M.); fulvio.morello@unito.it (F.M.); 2 Internal Medicine Unit, “Città della Salute e della Scienza di Torino, Molinette” University Hospital, 10126 Turin, Italy; betta.greco5@gmail.com; 3 Residency Program in Emergency Medicine, University of Turin, 10126 Turin, Italy; salvatoreandrea.randazzo@unito.it

**Keywords:** extracellular vesicles, microRNAs, COVID-19, bacterial sepsis

## Abstract

Severe COVID-19 and bacterial sepsis share clinical manifestations of systemic inflammation and organ dysfunction. Yet, early differentiation between these conditions and timely identification of patients at risk of deterioration remain major clinical challenges. Extracellular vesicle (EV)-associated microRNAs (miRNAs) have emerged as promising biomarkers of host immune dysregulation. In our study, we have characterized circulating EV-miRNAs in patients with COVID-19, bacterial sepsis, localized bacterial infections, and healthy subjects to assess their diagnostic and prognostic utility. After EV isolation from plasma and characterization by nanoparticle tracking analysis and flow cytometry, a panel of 12 inflammation-related miRNAs were individually quantified by qRT-PCR. Four EV-miRNAs—miR-28-5p, miR-199a-5p, miR-200a-3p, and miR-369-3p—were significantly elevated in COVID-19 patients, with higher levels in those with poor prognosis. miR-199a-5p and miR-200a-3p were increased in bacterial sepsis compared with COVID-19, enabling discrimination between viral and bacterial sepsis. Three EV-miRNAs—miR-28-5p, miR-199a-5p, and miR-200a-3p—were markedly higher in bacterial sepsis than localized infections, and ROC analysis showed a strong diagnostic performance, particularly for miR-199a-5p, alone or in combination with other EV-miRNAs. The increased expression of selected EV-miRNAs was associated with higher SOFA scores and in-hospital mortality. These findings indicate that EV-miRNAs reflect pathogen-specific and severity-related immune responses, supporting their potential as minimally invasive biomarkers for early diagnosis and risk stratification in severe infections.

## 1. Introduction

The Coronavirus Disease 2019 (COVID-19) pandemic has reshaped our understanding of sepsis, demonstrating that severe SARS-CoV-2 infection may fulfill the Sepsis-3 definition of viral sepsis, in which life-threatening organ dysfunction arises from a dysregulated host response to infection [1,2,3]. Importantly, not all patients with COVID-19 meet the criteria for viral sepsis; this diagnosis applies only to those who develop infection-related organ dysfunction. Moreover, many patients with severe COVID-19 develop secondary bacterial infections, which contribute to further increasing morbidity and mortality [4]. Clinical similarities between viral and bacterial sepsis can complicate the distinction between underlying causes of systemic inflammation [1,5] and in identifying patients at risk of rapid deterioration during the early phases of illness [1].

Sepsis remains a major global health burden, responsible for an estimated 49 million cases and 11 million deaths annually [6,7]. Early recognition and timely resuscitation are essential to improve outcomes; however, clinical features are often non-specific, and currently available biomarkers such as C-reactive protein and procalcitonin lack sufficient discriminatory ability, especially when differentiating viral from bacterial sepsis or localized from systemic infection [8,9]. Importantly, viral sepsis often elicits immunopathological patterns distinct from those observed in bacterial sepsis, including pronounced lymphopenia, elevated anti-inflammatory cytokines such as IL-10, and features of functional immunosuppression [10,11]. These differences underline the need for molecular biomarkers capable of capturing early and mechanistically relevant aspects of host dysregulation.

Although therapeutic strategies for COVID-19 and sepsis have evolved substantially, the field still faces critical unmet needs. Distinguishing viral from bacterial sepsis, recognizing the transition from localized to systemic infection, and predicting patient deterioration remain challenging with currently available biomarkers. Moreover, existing clinical scores capture organ dysfunction, but not necessarily the mechanisms of the underlying immunological dysregulation [2,12]. These gaps underscore the need for novel, mechanisms-based biomarkers capable of providing earlier and more specific insights into the immune host response.

Extracellular vesicles (EVs) and their RNA cargo have emerged as promising candidates for biomarkers in several diseases, including sepsis [13,14,15,16,17], as well as key mediators of intercellular communication and immune modulation during infection and inflammation [17,18]. EVs, released by virtually all cell types, carry microRNAs and other biomolecules that can influence recipient cell behavior locally or at distant sites [19]. EV-associated microRNAs (miRNAs)—short, approximately 22-nucleotide-long RNA molecules—regulate post-transcriptional gene expression and play pivotal roles in immune and inflammatory pathways [20]. Increasing evidence suggests that EV-miRNAs may provide a dynamic and integrative readout of the host response during severe infections, yet their comparative behavior in viral versus bacterial sepsis and their potential value for clinical risk assessment remain insufficiently explored [21].

The present study was designed as an observational, translational investigation aimed at identifying the diagnostic and prognostic value of EV-derived miRNAs (EV-miRNAs) in patients with COVID-19 and bacterial sepsis. By characterizing shared and disease-specific EV-miRNAs signatures and by integrating them with clinical outcomes, we sought to identify molecular indicators capable of improving the early discrimination between infection etiology and enhancing risk stratification in severe systemic inflammation, rather than to experimentally dissect the underlying molecular mechanisms of EV-miRNA action.

## 2. Results

### 2.1. Clinical Characteristics of Patients

We included 25 COVID-19 patients, 33 patients with bacterial sepsis, 23 patients with bacterial infection without sepsis, and 15 healthy subjects serving as controls. The mean age was comparable across the groups, and the gender distribution did not differ significantly (Table 1).

A higher prevalence of diabetes mellitus was observed in COVID-19 patients compared with the other groups. In contrast, comorbidities including hypertension and cancer were more common among patients with bacterial sepsis than in the other groups. Mean arterial pressure (MAP) was lower in patients with bacterial sepsis and the no-sepsis groups. In-hospital mortality occurred in 20% of COVID-19 patients and 21.2% of bacterial sepsis patients, while no deaths were recorded in the no-sepsis group. The Sequential Organ Failure Assessment (SOFA) score was significantly higher in the bacterial sepsis group compared with both COVID-19 and no-sepsis patients. The primary site of infection was respiratory (54.5%), gastrointestinal (12.1%), urinary tract (12.1%), and skin (15.1%) in patients with bacterial sepsis, whereas respiratory (39.4%), gastrointestinal (8.7%), and urinary tract (34.8%) in patients with localized infection. Detailed clinical characteristics for patients and controls are shown in Table 1.

White blood cell and platelet counts were higher in patients with bacterial sepsis compared with those with COVID-19, whereas hemoglobin levels were lower in infected groups than in healthy subjects. Markers of organ dysfunction and inflammation, including creatinine, lactate, bilirubin, NT-proBNP, and C-reactive protein, were significantly more elevated in patients with bacterial sepsis patients compared with the other groups. Procalcitonin was markedly increased in patients with bacterial sepsis than in those with COVID-19, consistent with bacterial infection. All the laboratory findings are summarized in Table 1.

### 2.2. Characterization of Extracellular Vesicles

According to NanoSight analysis, the particle size distribution showed a heterogeneous population with a modal diameter of approximately 200 nm (range: 100–400 nm). The size distribution and concentration remained consistent across samples, confirming the successful isolation of EVs. Flow cytometry analysis confirmed the presence of EV-specific surface markers, including CD9, CD63, and CD81, which are commonly associated with EV populations. Fluorescence intensity profiles displayed a clear shift compared to isotype controls, confirming the specificity of the staining. Overall, these results support the successful isolation and detailed characterization of EVs from plasma samples (Figure S1).

### 2.3. miRNA Expression in COVID-19 Patients: Comparison Between Patients with Good vs. Poor Prognosis

**Figure 1:**
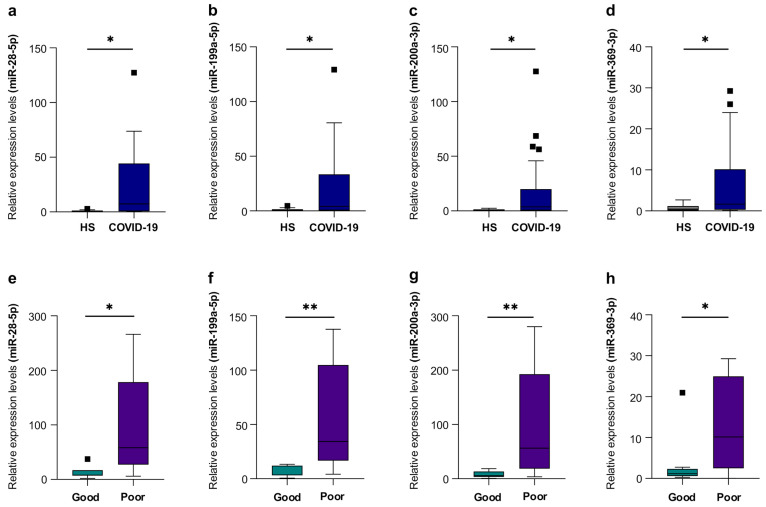
Relative expression levels of miR-28-5p (**a**), miR-199a-5p (**b**), miR-200a-3p (**c**), and miR-369-3p (**d**) in EVs isolated from plasma of HSs (*n* = 15) and COVID-19 patients (*n* = 25). Relative expression levels of miR-28-5p (**e**), miR-199a-5p (**f**), miR-200a-3p (**g**), and miR-369-3p (**h**) in EVs isolated from good-prognosis COVID-19 patients (*n* = 8) and poor-prognosis COVID-19 patients (*n* = 10). Data are presented as median (IQR), normalized with a reference exogenous control (Cel-miR-39). * *p* < 0.05, ** *p* < 0.01.

We studied the expression, in COVID-19 patients, of a panel of 12 miRNAs previously identified as critical regulator of inflammatory processes on the basis of functional data generated in our laboratory from bioinformatics analyses [22]. Out of these, four miRNAs—miR-28-5p, miR-199a-5p, miR-200a-3p, and miR-369-3p—were significantly higher in EVs from COVID-19 patients compared to healthy subjects (Figure 1). The remaining eight EV-miRNAs did not show statistically significant differences between groups.

We then studied these same miRNAs in COVID-19 patients with moderate symptoms at diagnosis, comparing patients who subsequently developed respiratory failure during hospitalization with those who did not, using respiratory failure as a clinical severity endpoint (Table S1). The levels of four miRNAs—miR-28-5p, miR-199a-5p, miR-200a-3p, and miR-369-3p—were higher in patients who developed respiratory failure (poor prognosis) than in those who did not (good prognosis) (Figure 1). These findings suggest that specific EV-associated miRNAs are not only elevated in COVID-19, but may also be linked to disease severity.

### 2.4. miRNA-EVs Aid Differential Diagnosis of COVID-19 and Bacterial Sepsis

**Figure 2:**
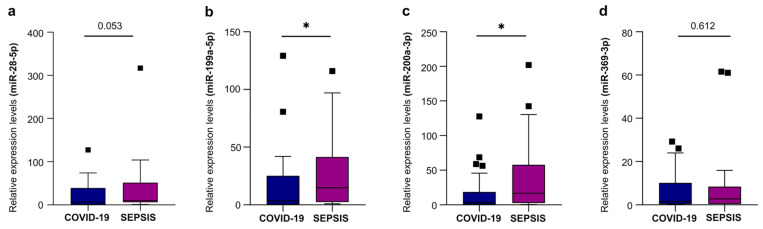
Relative expression levels of miR-28-5p (**a**), miR-199a-5p (**b**), miR-200a-3p (**c**), and miR-369-3p (**d**) in EVs isolated from plasma of COVID-19 patients (*n* = 25) and bacterial sepsis patients (*n* = 33). Data are presented as median (IQR), normalized with a reference exogenous control (Cel-miR-39). * *p* < 0.05.

When we evaluated the potential value of selected EV-miRNAs in discriminating between COVID-19 and bacterial sepsis, we found that the expression levels of two miRNA-EVs—miR-199a-5p and miR-200a-3p—were significantly higher in patients with bacterial sepsis compared with those with COVID-19 (Figure 2). On the contrary, miR-28-5p and miR-369-3p did not reach a statistically significant difference. This finding suggests that the two EV-miRNAs expression levels of these two EV-miRNAs may help in distinguishing COVID-19 from bacterial sepsis.

### 2.5. Diagnostic Value of EV-miRNAs in Bacterial Sepsis

The expression levels of the four EV-associated miRNAs previously identified as significantly upregulated in COVID-19 patients were also evaluated in patients with bacterial sepsis vs. patients with localized bacterial infections.

**Figure 3:**
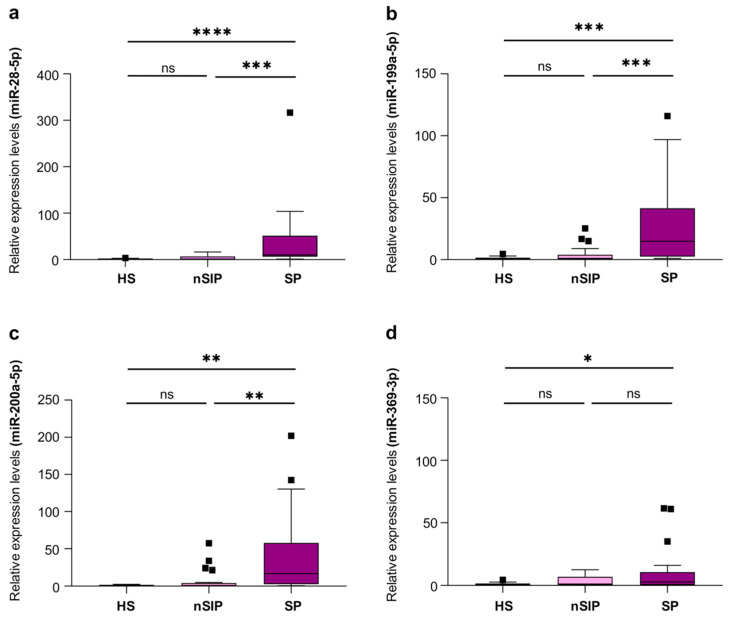
Relative expression levels of miR-28-5p (**a**), miR-199a-5p (**b**), miR-200a-3p (**c**), and miR-369-3p (**d**) in EVs isolated from plasma of HSs (*n* = 15), nSIP (*n* = 23), and SP (*n* = 33). Data are presented as median (IQR), normalized with a reference exogenous control (Cel-miR-39). HS, Healthy Subjects; nSIP, non-Septic Infected Patients; SP, Septic Patients; ns, not significant. * *p* < 0.05, ** *p* < 0.01, *** *p* < 0.0001, **** *p* < 0.00001.

All 4 EV-miRNAs—miR-28-5p, miR-199a-5p, miR-200a-3p, and miR-369-3p—showed increased expression in patients with bacterial sepsis compared with healthy controls. In addition, the levels of 3 EV-miRNAs—miR-28-5p, miR-199a-5p, miR-200a-3p—were higher also significantly more expressed in patients with bacterial sepsis than in those with localized bacterial infections (Figure 3).

**Figure 4:**
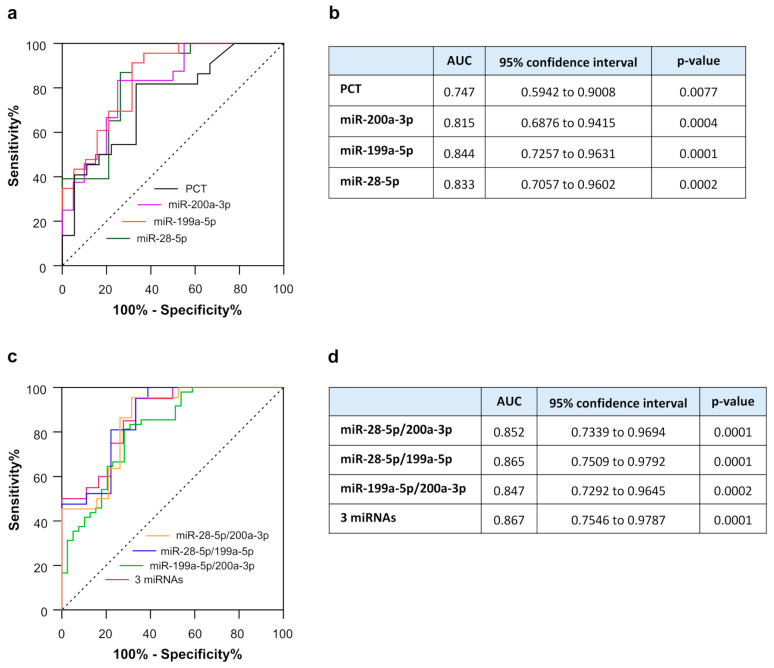
Receiver operating characteristic (ROC) curves of the candidate EV-miRNAs alone (**a**) and procalcitonin (PCT) for distinguishing between SP and nSIP. Panel (**a**): area under the curve (AUC); panel (**b**): discriminatory performance of each EV-miRNAs examined. Receiver operating characteristic (ROC) curves of the combined EV-miRNAs (**a**) for distinguishing between SP and nSIP. Panel (**c**): area under the curve (AUC); panel (**d**): discriminatory performance of each EV-miRNA examined.

In order to evaluate the diagnostic value of these EV-miRNAs, we generated a ROC curve. We analyzed only the statistically significant comparisons between septic and locally infected patients, based on our findings in Figure 3. For the three EV-miRNAs analyzed, miR-199a-5p presented the best individually discriminatory power in the comparison of sepsis vs. non-septic patients (Figure 4), presenting an area under the curve (AUC) of 0.844 (95% CI, 0.726–0.963; *p* = 0.0001), which outscored that of procalcitonin (PCT) [AUC = 0.747 (95% CI, 0.594–0.901; *p* = 0.0077)]. miR-28-5p and miR-200a-3p also showed a good diagnostic accuracy, with a ROC-AUC of 0.833 (95% CI, 0.706–0.960; *p* = 0.0002) and 0.815 (95% CI, 0.688–0.941; *p* = 0.0004), respectively.

Interestingly, the diagnostic accuracy resulted further improved when the expression levels of miR-199a-5p and miR-28-5p were combined, with a ROC-AUC value of 0.865 (95% CI, 0.729–0.979; *p* = 0.0001; Figure 4). Moreover, the combined ROC-AUC of miR-199a-5p, miR-28-5p, and miR-200a-3p slightly improved further to 0.867 (95% CI, 0.755–0.979; *p* = 0.0001; Figure 4). Taken together, these results indicate that a panel composed of two or three EV-miRNAs could be useful in diagnosing sepsis with high accuracy.

### 2.6. EV-miRNAs as Early Predictors of Bacterial Sepsis Severity

**Figure 5:**
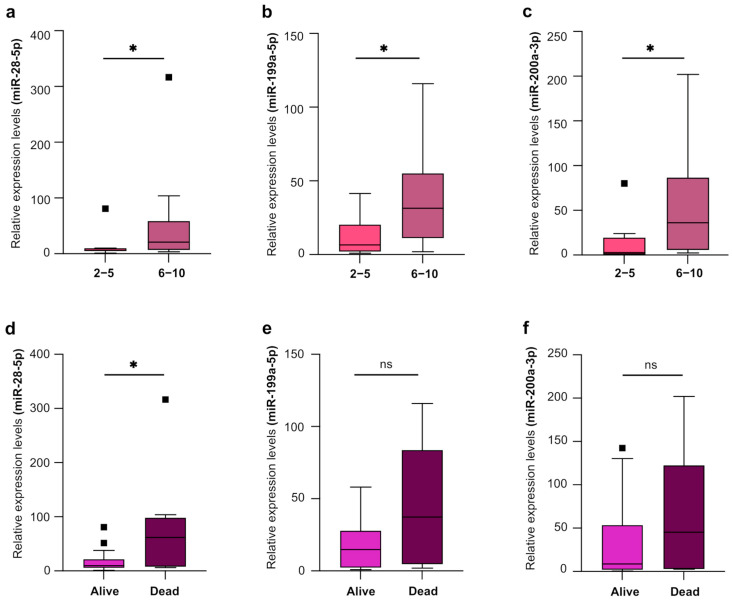
Relative expression levels of miR-28-5p (**a**), miR-199a-5p (**b**), and miR-200a-3p (**c**) in EVs isolated from plasma of patients with bacterial sepsis with 2–5 SOFA score (*n* = 17) and 6–10 SOFA score (*n* = 16). Relative expression levels of miR-28-5p (**d**), miR-199a-5p (**e**), and miR-200a-3p (**f**) in EVs isolated from plasma of alive (*n* = 26) and dead (*n* = 7) bacterial septic patients. Data are presented as median (IQR), normalized with a reference exogenous control (Cel-miR-39). ns, not significant. * *p* < 0.05.

The SOFA score, although frequently used in ICU patients, is quite complex as it requires the assessments of multiple parameters. When we analyzed the possible correlation between EV-miRNAs expression and SOFA score, we did not find any. However, after we stratified septic patients based on SOFA score severity into two groups (SOFA score = 2–5 and SOFA score = 6–10) (Table S2), we found that the expression of miR-199a-5p, miR-28-5p, and miR-200a-3p was higher in patients with higher compared to lower SOFA score (Figure 5).

Finally, when we analyzed septic patients based on in-hospital mortality (Table S3), we found a significant increase in the expression of has-miR-28-5p in those patients who died during their hospital stay (Figure 5d). On the contrary, the expression levels of miR-199a-5p and miR-200a-3p, although they showed a trend toward an increase in septic patients who died, did not reach the statistical significance.

## 3. Discussion

Severe COVID-19 has underscored the extent to which viral infections can trigger life-threatening organ dysfunction, aligning with the current definition of sepsis [2] and highlighting the blurred boundary between viral and bacterial systemic inflammation. Despite substantial progress in the management of both COVID-19 and bacterial sepsis [1,23], early clinical differentiation and accurate risk stratification of disease severity [1,5] remain challenging in a subset of patients. Commonly used biomarkers, such as C-reactive protein and procalcitonin, often lack specificity, while clinical scoring systems typically reflect downstream consequences of dysregulated host responses rather than their early molecular drivers [24,25,26]. Recent studies emphasize the need for mechanism-based biomarkers capable of capturing upstream immune and endothelial perturbations that precede overt clinical deterioration [27,28]. Within this framework, extracellular vesicles (EVs) and their miRNA cargo have emerged as promising candidates, owing to their stability, molecular complexity, and ability to mirror real-time cellular responses during infection and inflammation [29,30].

In this study, among the twelve circulating miRNAs previously associated with inflammatory pathways in COVID-19 [22], four—miR-28-5p, miR-199a-5p, miR-200a-3p, and miR-369-3p—were selectively enriched in plasma-derived extracellular vesicles from COVID-19 patients. Notably, their expression levels were further increased in individuals with poor clinical outcomes compared with those with favorable prognosis. These findings support the hypothesis that EV-derived miRNAs act as sensitive indicators of immune dysfunction in viral infections and may reflect early molecular events that precede overt clinical deterioration.

When we extend the evaluation of individual EV-miRNAs to patients with bacterial sepsis as well, two EV-miRNAs—miR-199a-5p and miR-200a-3p—resulted in markedly higher levels than in COVID-19 patients, a pattern consistent with the profound endothelial dysfunction, metabolic derangement, and oxidative stress that characterize bacterial sepsis [31,32]. In contrast, miR-28-5p and miR-369-3p did not differ significantly between the two groups; however, both have been previously associated with interferon signaling and antiviral responses [33,34,35], which aligns with their selective elevation in COVID-19 [34]. The different number of EV-miRNAs expressed and altered may reflect differences in the underlying disease mechanisms and pathophysiological processes present in COVID-19 and in bacterial sepsis [27]. Our study yielded two key findings: (1) EV-derived miRNAs differed markedly between COVID-19 and bacterial sepsis, with two miRNAs showing disease-specific expression patterns, and (2) three miRNAs emerged as potential biomarkers useful for distinguishing sepsis from localized infection and for predicting adverse clinical outcomes.

Our findings build upon previous observations that circulating miRNAs are altered during systemic infections [36,37,38]. However, whereas earlier studies often reported broad and heterogeneous miRNA dysregulation in COVID-19 or sepsis [39,40], we observed a much more selective modulation restricted to four EV-associated miRNAs. This aligns with emerging evidence that EV loading is not random, but it is rather shaped by pathogen-specific signaling pathways [41].

Together, these pathogen-specific expression patterns reinforce the concept that EV-miRNA release is not merely a by-product of systemic inflammation but reflects targeted regulatory programs activated by distinct infectious stimuli.

Our findings partially align with previous studies reporting that miR-199a-5p is present in bronchial aspirate in critically ill COVID-19 patients and could be used as a marker to distinguish between COVID-19 and non-COVID-19 patients [42]. On the other hand, miR-199a-5p plays a complex role in bacterial sepsis, primarily acting to worsen intestinal barrier dysfunction, promote inflammation (via NF-kB), and increase organ damage by targeting protective factors, contributing to the severity of sepsis [43]. However, it should be underlined that our study does not provide direct experimental evidence of the molecular mechanisms through which EV-associated miR-199a-5p modulated inflammatory pathways. The mechanistic roles described above have been described in previous functional studies and should be interpreted in the context of our findings as biologically plausible associations rather than causal relationships. Functional in vitro and in vivo studies will be required to directly investigate the role of EV-associated miR-199a-5p in immune regulation and to link EV-miRNA expression differences to specific molecular pathways.

Previous research has shown that the miR-200 family expression, limited to miR-200c-3p, is upregulated in saliva [44] and serum [45] of COVID-19 patients [46].

Current evidence supports that SOFA scores may be considered as a valuable tool for predicting mortality in critically ill COVID-19 patients [47,48]; however, relying solely on the SOFA score appears insufficient to fully characterize disease severity in this population [47,48]. Patients with severe COVID-19 often present heart, kidney, or liver dysfunction in addition to respiratory failure [49]. These clinical features are strictly related to the concept of sepsis-related multi-organ dysfunction and significantly contribute to clinical deterioration and mortality in the most severe cases. In our study, COVID-19 patients showed significantly lower SOFA score compared with bacterial sepsis, despite presenting signs of organ dysfunction.

The symptoms and signs of sepsis lack specificity, making diagnosis, treatment, and prognosis evaluation challenging. Identifying biomarkers capable of reliably distinguishing sepsis from non-septic infection remains a major challenge, as many inflammatory markers rise in both conditions and lack sufficient specificity [50]. In our study, the diagnostic potential of EV-miRNAs was further supported by their ability to discriminate bacterial sepsis from localized infections. Specifically, three miRNAs—miR-28-5p, miR-199a-5p, and miR-200a-3p—were significantly higher in patients with sepsis compared with patients with localized infection, indicating that their expression was associated with systemic, rather than localized, inflammatory responses and organ damage development. Importantly, miR-199a-5p exhibited superior diagnostic accuracy and the best individual diagnostic performance, outperforming procalcitonin, a routinely used biomarker in sepsis assessment. This may suggest that EV-miRNAs may act as upstream indicators of pathophysiological imbalance rather than downstream inflammatory by-products. The improved performance of combined miRNAs reinforces the concept that sepsis is a multifactorial syndrome requiring multidimensional biomarkers for optimal detection.

The function of miR-28-5p is highly versatile and is generally described as tumor-suppressive, inhibiting cell proliferation, migration, and invasion in several cancers (including breast and renal) by targeting oncogenes like RAP1B, WSB2, and SphK1 [51,52]. However, no evidence is currently available regarding its role in sepsis or systemic infections.

miR-199a-5p and miR-200a-3p play a crucial role in bacterial sepsis. Specifically, miR-199a-5p is shown to be dysregulated in neonatal sepsis, hinting at its involvement in the immune responses [53]. miR-200a-3p plays a pro-inflammatory role in bacterial sepsis, particularly in brain injury, by promoting the NLRP3 inflammasome pathway, increasing reactive oxygen species (ROS), and suppressing the anti-inflammatory Keap1/Nrf-2/HO-1 axis [54]. This could make miR-200a-3p a potential candidate biomarker and therapeutic target in sepsis, although its exact function in bacterial sepsis needs more exploration [43,54].

To further explore the prognostic relevance of these EV-miRNAs, we examined their association with clinical severity and outcomes. Although we found no direct correlation between miRNA expression and SOFA score, high levels of miR-199a-5p, miR-28-5p, and miR-200a-3p were observed in patients with higher SOFA scores. This suggests that EV-miRNAs may reflect biological dimensions of organ dysfunction not fully captured by clinical scoring systems. The prognostic value of miR-28-5p for in-hospital mortality further supports the potential of EV-miRNAs as early indicators of adverse outcomes in sepsis, complementing existing clinical tools.

Nevertheless, the results of our study should be interpreted while taking into account several limitations. First, this was a single-center study involving a relatively small patient cohort, which may limit the generalizability of the results. Second, the moderate sample size and cross-sectional design do not allow for the evaluation of temporal changes during disease progression, since sample collection at a unique time point following COVID-19 or sepsis diagnosis prevented the evaluation of temporal trajectories in EV-miRNAs expression. Third, although the identified EV-miRNAs showed promising diagnostic and prognostic potential, their clinical relevance and stability across different patient populations require validation in larger, multicenter cohorts and through longitudinal analyses. In addition, this study was not designed to experimentally investigate the molecular mechanisms by which EV-associated miRNAs influence the clinical course of sepsis. Therefore, while we report associations with clinical outcomes, causality and specific functional roles remain to be established. Future in vitro and in vivo studies are needed to elucidate the underlying pathways and link expression differences to clinical significance. Finally, EV characterization was based on flow cytometric detection of canonical tetraspanin markers, while ultrastructural analysis by transmission electron microscopy was not performed, representing a technical limitation of the study.

Overall, our findings reveal that EV-associated miRNAs carry discriminative and clinically meaningful information, enabling differentiation between infection types and reflecting the severity of critical infectious diseases. These insights position EV-miRNAs as readily accessible, mechanistically informative biomarkers with strong translational potential for enhancing diagnostic precision, early risk assessment, and therapeutic decision-making in severe infectious diseases.

## 4. Materials and Methods

### 4.1. Patients

A prospective observational study was carried out at the Emergency Department of the “Città della Salute e della Scienza di Torino—Molinette Site” University Hospital, Turin, Italy, from February 2021 to September 2025. The study received approval from the Institutional Ethics and Review Board of the “Città della Salute e della Scienza di Torino” University Hospital, Turin, Italy (n. CS2/815), and adhered to the ethical standards outlined by the Declaration of Helsinki and its subsequent amendments. Written informed consent was obtained from all patients before enrolment.

#### 4.1.1. COVID-19 Patients

Adults (age ≥ 18 years) presenting to the Emergency Department (ED) with symptoms suggestive of SARS-CoV-2 infection (i.e., fever, dyspnea, cough, sore throat, diarrhea, ageusia, anosmia) were screened for inclusion. SARS-CoV-2 infection was confirmed by qRT-PCR testing on nasopharyngeal swab samples collected at ED admission. Patients were excluded if they died or required orotracheal intubation within 24 h of admission, or if they had malignancies under active treatment. Final case adjudication into COVID-19 positive or negative was independently performed by two expert physicians based on all available clinical and laboratory data; disagreements were resolved by a third clinician.

COVID-19 patients were further stratified according to disease severity during hospitalization: (i) *good prognosis*: patients with moderate symptoms at enrolment who did not require oxygen therapy; (ii) *poor prognosis*: patients with moderate symptoms at enrolment who subsequently required oxygen therapy, continuous positive airway pressure (CPAP), or admission to the intensive care unit (ICU) with mechanical ventilation. This stratification was used to describe clinical disease progression and was not intended to define viral sepsis. Respiratory support requirements were utilized as clinical outcomes rather than as criterium for sepsis diagnosis. At the time of first medical evaluation in the ED, SOFA score was comparable between groups and viral sepsis was not inferred from respiratory support requirements alone.

#### 4.1.2. Bacterial Sepsis Patients and Controls

Patients presenting to the ED with clinical signs and symptoms indicative of bacterial infection and meeting the Sepsis-3 criteria [2] for sepsis (suspected or confirmed infection accompanied by an increase in SOFA scores > 2) were included in the bacterial sepsis group. Exclusion criteria included the following: age < 18 years, refusal to participate, alternative non-infectious diagnoses (e.g., trauma), the unavailability of plasma samples collected at presentation, autoimmune disease, or current use of immunosuppressive drugs.

Bacterial sepsis groups were stratified according to mean SOFA scores into two groups (SOFA 2–5; SOFA 6–10) and additionally classified based on in-hospital moratlity (alive vs. dead).

Patients with a localized bacterial infection but without sepsis (i.e., no evidence of end-organ dysfunction, shock, or SOFA score change < 2) were also enrolled and classified as the control group of septic patients. Diagnosis was based on clinical presentation, laboratory findings, and microbiological confirmation.

Healthy subjects (HS) were recruited from laboratory staff members and served as an additional control group.

### 4.2. Sample Collection

Blood samples were collected into 9 mL EDTA-coated tubes (BD Vacutainer, Reading, UK) using a central venous catheter or, in healthy subjects, by clean venipuncture using a 21-gauge butterfly infusion set and processed within 1 h of collection. Samples were first centrifuged at 1500× *g* for 10 min at 4 °C to remove cellular components and promptly stored at −80 °C until subsequent analysis.

### 4.3. EV Isolation by Charge-Based Precipitation Method

EVs were isolated by charge-based precipitation method, as previously described [16]. Approximately 1 mL of plasma from patients and controls was centrifuged at 5000× *g* at 4 °C for 30 min to remove cellular debris and platelet contamination. Briefly, a protamine (P) (Sigma, St. Louis, MO, USA)/Polyethylene glycol (PEG 35,000; Merck KGaA, Darmstadt, Germany) precipitation solution (P/PEG; Sigma, St. Louis, MO, USA) (0.2 g PEG 35,000 an 1 mg protamine chloride/mL; 1:4) was added to the plasma samples. After overnight incubation at 4 °C, the mixture was centrifuged at 1500× *g* for 30 min at 4 °C, followed by a second centrifugation at 1500× *g* for 30 min at 4 °C. The pellet was suspended in 150 μL of PBS-free particles and stored at −80 °C for subsequent RNA extraction.

### 4.4. EV Characterization

For EV characterization, we have followed the recommendations from the Minimal Information for Studying Extracellular Vesicles (MISEV) 2023 guidelines [55].

#### 4.4.1. Nanoparticle Tracking Analysis

Particle concentration and size were measured using nanoparticle tracking analysis (NTA) with a NanoSight LM10 instrument equipped with a 405 nm laser and Nanosight Tracking Analyses 2.3 software (Malvern Panalytical Ltd., Malvern, UK). Briefly, EVs were diluted 1:1000 in particle-free PBS before analysis. Three consecutive 30 s recordings were obtained per sample. The minimum expected particle size, minimum track length, and biomedical light unit setting were set to automatic, while the detection threshold was set to 4 to detect all particles.

#### 4.4.2. Flow Cytometer Analysis

Expression of specific surface markers in EVs was evaluated by flow cytometry. Twenty microliters of EVs were labeled with either a phycoerythrin (PE; Invitrogen, ThermoFisher Scientific, Carlsbad, CA, USA)-conjugated mouse anti-human IgG1 monoclonal antibody against the tetraspanins CD9 (clone eBioSN4, C3-3A2), a PE-Cyanine7 (Invitrogen, ThermoFisher Scientific)-conjugated mouse anti-human IgG1 monoclonal antibody against the tetraspanins CD63 (clone H5C6), or a PerCP-eFluor 710-conjugate antibody against CD81 (clone 1D6-CD81; Invitrogen, ThermoFisher Scientific) at a volume of 5 µL/test in a final volume of 100 µL of diluted particle-free PBS, for 1 h at room temperature in the dark. Mouse IgG1 kappa isotype control monoclonal antibodies lacking specificity for CD9, CD63, or CD81 (P3.6.2.8.1, PE or PE-Cyanine7, Invitrogen, ThermoFisher Scientific) were used to measure the signal from non-specific flow cytometry interactions. The Attune NxT Small Particle Side-Scatter Filter (488/10) (Invitrogen, ThermoFisher Scientific) was installed to enable SSC resolution at the scale required to visualize nanoparticles in the Attune NxT Acoustic Focusing Flow Cytometer system (ThermoFisher Scientific), as previously described [17].

### 4.5. Determination and Isolation of EV-miRNAs

Based on a previous study [22], we have chosen 12 EV-miRNAs identified for their associations with inflammatory pathways and which we found to be upregulated in patients with COVID-19 compared to healthy controls. These included hsa-miR-100-5p (478224_mir), hsa-miR-106b-5p (478412_mir), hsa-miR-133a-3p (478511_mir), hsa-miR-142-3p (477910_mir), hsa-miR-151b (477811_mir), hsa-miR-199a-5p (478231_mir), hsa-miR-200a-3p (478490_mir), hsa-miR-28-5p (478000_mir), hsa-miR-369-3p (478067_mir), has-miR-374a-5p (478238_mir), hsa-miR-423-3p (478327_mir), and hsa-miR-545-3p (479002_mir). The synthetic cel-miR-39-3p (478293_mir) (Applied Biosystems, ThermoFisher Scientific) was used as an exogenous control to monitor RNA extraction efficiency.

miRNAs were isolated from 150 µL of EV pellet using miRNeasy Mini Kit (Qiagen, Crawley, UK) following the manufacturer’s instructions. Spike-in control Cel-miR-39-3p (Applied Biosystems, ThermoFisher Scientific) from Caenorhabditis elegans solution (1.6 × 10^8^ copies/µL) was added as an exogenous control to assess RNA extraction efficiency. RNA, predominantly miRNAs and other small RNAs, was collected in a final elution volume of 20 µL of RNase-free water and stored at −80 °C.

### 4.6. Reverse Transcription

First, 2 µL of miRNA extracts was reverse-transcribed into complementary DNA (cDNA) using the TaqMan Advanced miRNA cDNA Synthesis Kit (Applied Biosystems, ThermoFisher Scientific) according to the manufacturer’s instructions. Briefly, 5 µL of reverse transcription product was used for pre-amplification with a custom miR-Amp Primer Mix and the miR-Amp Master Mix (Applied Biosystems, ThermoFisher Scientific). Finally, 50 µL of pre-amplified cDNA was stored at −20 °C until further analysis, which was conducted within two months.

### 4.7. qRT-PCR

We performed single-tube assays on individual samples from each patient enrolled in the study using a QuantStudio 1 Real-Time PCR System (Applied Biosystems, ThermoFisher Scientific). Pre-amplified cDNA samples were diluted 1:10 in 0.1× TE buffer. Briefly, Master Mix, assays, water, and diluted pre-amplified cDNA samples were placed in individual wells of an optical 96-well reaction plate. Each reaction was conducted in triplicate. The primers used are listed in Table S3. Samples were run using the following parameters: 50 °C for 2 min, 95 °C for 10 min, followed by 40 cycles of 95 °C for 15 s and 60 °C for 1 min, with a final hold at 4 °C.

The relative miRNA expression was calculated using the comparative cycle threshold (2^−ddCt^) method, normalized to cel-miR-39-3p levels (exogenous controls).

All calculations were performed using the QuantStudio Real-Time PCR Software v1.3 (Applied Biosystems, ThermoFisher Scientific).

### 4.8. Statistical Analysis

Descriptive statistics were presented as median (interquartile range, IQR) or mean (±standard error of the mean, SEM), depending on data distribution as evaluated by the Shapiro–Wilk test. Group comparisons were performed using a Kruskal–Wallis one-way analysis of variance on ranks followed by Dunn’s multiple comparison tests, or unpaired or paired Student’s *t*-test, as suitable. The diagnostic accuracy of each miRNA was calculated as the area under the receiver operating characteristic curve (AUC–ROC).

A *p*-value < 0.05 was deemed significant.

Data were collected with an Excel spreadsheet (Microsoft Office 365 ProPlus), and analyses were carried out using GraphPad Prism 9.0 software for Windows and Macintosh (GraphPad Software, La Jolla, CA, USA).

## Figures and Tables

**Table 1:** Demographic and clinical characteristics of patients and healthy subjects.

Characteristics	Healthy Subjects (HS, *n* = 15)	COVID-19 Patients (COVID-19, *n* = 25)	Non-Septic Infected Patients (nSIP, *n* = 23)	Bacterial Sepsis (SP, *n* = 33)	*p*-Value
Age, year, mean	61 (±19.61)	70.5 (±13.88)	67.1 (±20.5)	74.48 (±12.99)	0.152
Gender ratio (M/F)	8/7	15/10	14/9	17/16	0.874
Comorbidities					
Diabetes Mellitus, *n* (%)	0	14 (56.00%)	6 (26.09%)	9 (28.12%)	0.002
Hypertension, *n* (%)	0	16 (64.00%)	9 (39.13%)	20 (62.50%)	0.002
Obesity, *n* (%)	0	5 (20.00%)	2 (8.70%)	5 (15.62%)	0.263
Cancer, *n* (%)	0	0	2 (8.70%)	7 (21.2%)	0.008
Smoking, *n* (%)	2 (13.33%)	2 (8.00%)	2 (8.70%)	5 (15.62%)	0.778
MAP	-	116.9 (±18.53)	94.50 (±15.51)	90.60 (±15.31)	<0.0001
In-hospital death, *n* (%)	0	5 (20%)	0	7 (21.2%)	0.029
SOFA score	-	2 (2–3)	0 (0–0) ***	5.54 (±2.39) ***^§§§§^	<0.0001
Primary Site of Infections, *n* (%)					
Pneumonia (Respiratory)	-	25 (100%)	13 (39.4%)	18 (54.5%)	<0.0001
Abdominal (Gastrointestinal)	-	0	2 (8.7%)	6 (12.1%)	0.184
Urinary tract	-	0	8 (34.8%)	4 (12.1%)	0.001
Skin	-	0	0	5 (15.1%)	0.018
Laboratory Findings					
White Blood Cell (10^9^/L)	5.13 (±0.55)	6.73 (4.84–8.67)	8.04 (5.65–12.45)	10.04 (6.12–14.02) *°	0.007
Hemoglobin (g/dL)	13.58 (±0.13)	13.50 (±1.66)	11.27 (±2.09) **	12.30 (9.95–13.95)	0.007
Platelets (10^9^/L)	210.7 (±46.42)	187.5 (153.3–241.5)	228.4 (±72.43)	227 (131–302)	0.800
Creatinine (mg/dL)	0.86 (±0.12)	0.89 (0.80–1.11)	0.74 (0.38–1.22)	1.64 (1.00–2.55) *^§§§^	0.0004
Bilirubin (mg/dL)	-	0.60 (0.40–0.80)	0.5 (0.3–0.8)	1.10 (0.55–2) *^§§^	0.004
Lactate (mmol/L)	-	1.10 (0.80–1.50)	1.25 (0.9–2.07)	2.35 (1.05–5.60) **	0.005
C-Reactive Protein (mg/L)	3.15 (±2.17)	80.51 (±55.98) °°	100 (76.10–154.3) °°°	91 (31.40–164.2) °°°	0.0002
Procalcitonin (ng/mL)	0.02 (0.02–0.02)	0.09 (0.06–0.18) °°	0.22 (0.07–2.03) °°°°	0.64 (0.14–7.19) °°°°**	<0.0001

Mean (±SD) or median (IQR) as appropriate. * vs. COVID-19 *p*-value < 0.05; ** COVID-19 *p*-value < 0.001; *** vs. COVID-19 *p*-value < 0.0001. ^§§^ COVID-19 *p*-value < 0.001; ^§§§^ vs. COVID-19 *p*-value < 0.0001; ^§§§§^ vs. COVID-19 *p*-value < 0.00001. ° vs. HS *p*-value < 0.05; °° vs. HS *p*-value < 0.0001; °°° vs. HS *p*-value < 0.0001; °°°° vs. HS *p*-value < 0.00001.

## Data Availability

Deidentified participant data will be made available on a collaborative basis upon reasonable request. Data and research materials used in this study are available upon request to qualified researchers for purposes of replication, further analysis, and academic collaboration.

## Supplementary Materials

The following supporting information can be downloaded at https://www.mdpi.com/article/10.3390/ijms27031334/s1.

## References

[1] Koçak Tufan Z., Kayaaslan B., Mer M. (2021). COVID-19 and Sepsis. Turk. J. Med. Sci..

[2] Singer M., Deutschman C.S., Seymour C.W., Shankar-Hari M., Annane D., Bauer M., Bellomo R., Bernard G.R., Chiche J.-D., Coopersmith C.M. (2016). The Third International Consensus Definitions for Sepsis and Septic Shock (Sepsis-3). JAMA.

[3] Herminghaus A., Osuchowski M.F. (2022). How Sepsis Parallels and Differs from COVID-19. eBioMedicine.

[4] Wu G., Lu J., Liu D., He Y. (2023). Characteristics and Risk Factors of Secondary Bacterial Infections in COVID-19 Patients. Antimicrob. Steward. Healthc. Epidemiol..

[5] Ren C., Yao R.-Q., Ren D., Li Y., Feng Y.-W., Yao Y.-M. (2020). Comparison of Clinical Laboratory Tests Between Bacterial Sepsis and SARS-CoV-2-Associated Viral Sepsis. Mil. Med. Res..

[6] Rudd K.E., Johnson S.C., Agesa K.M., Shackelford K.A., Tsoi D., Kievlan D.R., Colombara D.V., Ikuta K.S., Kissoon N., Finfer S. (2020). Global, Regional, and National Sepsis Incidence and Mortality, 1990–2017: Analysis for the Global Burden of Disease Study. Lancet.

[7] La Via L., Sangiorgio G., Stefani S., Marino A., Nunnari G., Cocuzza S., La Mantia I., Cacopardo B., Stracquadanio S., Spampinato S. (2024). The Global Burden of Sepsis and Septic Shock. Epidemiologia.

[8] Wang N., Huang H., Tan Y., Zhang N. (2025). Research Progress of Biomarkers for Sepsis and Precision Medicine. Emerg. Med. Int..

[9] Daud M., Khan M.B., Qudrat Q.U., Ullah I., Khan S., Khan M.Z., Yousuf I., Ahmad F. (2024). Role of C-Reactive Protein and Procalcitonin in Early Diagnostic Accuracy and Their Prognostic Significance in Sepsis. Cureus.

[10] Saavedra-Torres J.S., Pinzón-Fernández M.V., Nati-Castillo H.A., Cadena Correa V., Lopez Molina L.C., Gaitán J.E., Tenorio-Castro D., Lucero Guanga D.A., Arias-Intriago M., Tello-De-la-Torre A. (2025). Immunodynamic Disruption in Sepsis: Mechanisms and Strategies for Personalized Immunomodulation. Biomedicines.

[11] Xiong L., Tang H., Xie Q., Fang H., Jing D., Chen L. (2025). Immune Signatures Distinguish Pure and Mixed Sepsis in Critical COVID-19: A Retrospective Cohort Study. J. Inflamm. Res..

[12] Castello L.M., Gavelli F. (2024). Sepsis Scoring Systems: Mindful Use in Clinical Practice. Eur. J. Intern. Med..

[13] Im Y., Yoo H., Ko R.-E., Lee J.Y., Park J., Jeon K. (2021). Exosomal CD63 in Critically Ill Patients with Sepsis. Sci. Rep..

[14] Dakhlallah D.A., Wisler J., Gencheva M., Brown C.M., Leatherman E.R., Singh K., Brundage K., Karsies T., Dakhlallah A., Witwer K.W. (2019). Circulating Extracellular Vesicle Content Reveals de Novo DNA Methyltransferase Expression as a Molecular Method to Predict Septic Shock. J. Extracell. Vesicles.

[15] O’Toole H.J., Lowe N.M., Arun V., Kolesov A.V., Palmieri T.L., Tran N.K., Carney R.P. (2024). Plasma-Derived Extracellular Vesicles (EVs) as Biomarkers of Sepsis in Burn Patients via Label-Free Raman Spectroscopy. J. Extracell. Vesicles.

[16] Schiavello M., Bosco O., Vizio B., Sciarrillo A., Pensa A., Pivetta E., Morello F., Risso D., Montrucchio G., Mariano F. (2025). Profiling of miRNAs Contained in Circulating Extracellular Vesicles and Associated with Sepsis Development in Burn Patients: A Proof-of-Concept Study. Int. J. Mol. Sci..

[17] Schiavello M., Vizio B., Bosco O., Mariano F., Bruno S., Pensa A., Cagna Vallino P., Dini C., Montrucchio G., Lupia E. (2025). CD42-Enriched Extracellular Vesicles Contribute to Increased Platelet Aggregation and Possibly Organ Damage in Patients with Burn Injury Complicated by Sepsis. Int. J. Nanomed..

[18] Repici A., Piraino G., Wolfe V., Kaplan J., Nakamura T., Zingarelli B., Repici A., Piraino G., Wolfe V., Kaplan J. (2025). Role of Extracellular Vesicles as Mediators of Cell Communication and Novel Biomarkers in Sepsis. J. Clin. Med..

[19] Kumar M.A., Baba S.K., Sadida H.Q., Marzooqi S.A., Jerobin J., Altemani F.H., Algehainy N., Alanazi M.A., Abou-Samra A.-B., Kumar R. (2024). Extracellular Vesicles as Tools and Targets in Therapy for Diseases. Signal Transduct. Target. Ther..

[20] Bartel D.P. (2004). MicroRNAs: Genomics, Biogenesis, Mechanism, and Function. Cell.

[21] Yoshikawa F.S.Y., Teixeira F.M.E., Sato M.N., Oliveira L.M.d.S. (2019). Delivery of microRNAs by Extracellular Vesicles in Viral Infections: Could the News Be Packaged?. Cells.

[22] Schiavello M., Vizio B., Sanavia T., Bosco O., Dini C., Vallino P.C., Pivetta E., Morello F., Fariselli P., Montrucchio G. (2025). Utility of Plasma MicroRNA Profiling as Diagnostic Biomarker in Immune System Activation and Inflammation and Early Predictor of Severity in Patients with COVID-19. Sci. Rep..

[23] Cavaillon J.-M., Chousterman B.G., Skirecki T. (2024). Compartmentalization of the Inflammatory Response during Bacterial Sepsis and Severe COVID-19. J. Intensive Med..

[24] Xinyu X., Jiang Z., Qing A., Lihua L., Xiehong L., Lin Z. (2025). Clinical Significance of PCT, CRP, IL-6, NLR, and TyG Index in Early Diagnosis and Severity Assessment of Acute Pancreatitis: A Retrospective Analysis. Sci. Rep..

[25] Xu Z., Zhang J., Li Z., Wu H., Xu H., Guo Y., Li Y. (2025). Organ-Targeted Biomarkers of Sepsis: A Systematic Review Reveals the Value of Inflammation and Lipid Metabolic Dysregulation. Pharmacol. Res..

[26] Nargis W., Ibrahim M., Ahamed B.U. (2014). Procalcitonin versus C-Reactive Protein: Usefulness as Biomarker of Sepsis in ICU Patient. Int. J. Crit. Illn. Inj. Sci..

[27] Muratsu A., Oda S., Onishi S., Yoshimura J., Matsumoto H., Togami Y., Mitsuyama Y., Ito H., Okuzaki D., Ogura H. (2024). Bacterial Sepsis Causes More Dramatic Pathogenetic Changes in the Th1 Pathway than Does Viral (COVID-19) Sepsis: A Prospective Observational Study of Whole Blood Transcriptomes. Virol. J..

[28] Dong X., Wang C., Liu X., Gao W., Bai X., Li Z. (2020). Lessons Learned Comparing Immune System Alterations of Bacterial Sepsis and SARS-CoV-2 Sepsis. Front. Immunol..

[29] Xu D., Di K., Fan B., Wu J., Gu X., Sun Y., Khan A., Li P., Li Z. (2022). MicroRNAs in Extracellular Vesicles: Sorting Mechanisms, Diagnostic Value, Isolation, and Detection Technology. Front. Bioeng. Biotechnol..

[30] Sanz-Rubio D., Martin-Burriel I., Gil A., Cubero P., Forner M., Khalyfa A., Marin J.M. (2018). Stability of Circulating Exosomal miRNAs in Healthy Subjects. Sci. Rep..

[31] Vincent J.-L., Ince C., Pickkers P. (2021). Endothelial Dysfunction: A Therapeutic Target in Bacterial Sepsis?. Expert Opin. Ther. Targets.

[32] Ghazal P., Rodrigues P.R.S., Chakraborty M., Oruganti S., Woolley T.E. (2022). Challenging Molecular Dogmas in Human Sepsis Using Mathematical Reasoning. eBioMedicine.

[33] Han Y., Zhang G., Lv X., Ren L. (2025). Critical Role of Cellular microRNAs in Virus Infection: Decades of Progress. Anim. Zoonoses.

[34] Roustai Geraylow K., Hemmati R., Kadkhoda S., Ghafouri-Fard S. (2022). miRNA Expression in COVID-19. Gene Rep..

[35] Mustafa F., Ahmad W., Gull B., Baby J., Panicker N.G., Khader T.A., Baki H.A., Rehman E., Salim A.M., Ahmed R.L.G. (2025). miRNA Biomarkers for Prognosis and Therapy Monitoring in a Multi-Ethnic Cohort with SARS-CoV-2 Infection. Sci. Rep..

[36] Mi S., Zhang J., Zhang W., Huang R.S. (2013). Circulating MicroRNAs as Biomarkers for Inflammatory Diseases. MicroRNA.

[37] Van der Auwera S., Ameling S., Wittfeld K., Bülow R., Nauck M., Völzke H., Völker U., Grabe H.J. (2024). Circulating miRNAs Modulating Systemic Low-Grade Inflammation and Affecting Neurodegeneration. Prog. Neuropsychopharmacol. Biol. Psychiatry.

[38] Garcia-Giralt N., Du J., Marin-Corral J., Bódalo-Torruella M., Blasco-Hernando F., Muñoz-Bermúdez R., Clarós M., Nonell L., Perera-Bel J., Fernandez-González M. (2022). Circulating microRNA Profiling is Altered in the Acute Respiratory Distress Syndrome Related to SARS-CoV-2 Infection. Sci. Rep..

[39] Mortazavi-Jahromi S.S., Aslani M. (2022). Dysregulated miRNAs Network in the Critical COVID-19: An Important Clue for Uncontrolled Immunothrombosis/Thromboinflammation. Int. Immunopharmacol..

[40] Formosa A., Turgeon P., dos Santos C.C. (2022). Role of miRNA Dysregulation in Sepsis. Mol. Med..

[41] Dixson A., Dawson T.R., Di Vizio D., Weaver A.M. (2023). Context-Specific Regulation of Extracellular Vesicle Biogenesis and Cargo Selection. Nat. Rev. Mol. Cell Biol..

[42] Molinero M., Benítez I.D., González J., Gort-Paniello C., Moncusí-Moix A., Rodríguez-Jara F., García-Hidalgo M.C., Torres G., Vengoechea J.J., Gómez S. (2022). Bronchial Aspirate-Based Profiling Identifies MicroRNA Signatures Associated with COVID-19 and Fatal Disease in Critically Ill Patients. Front. Med..

[43] Du X., Tian D., Wei J., Yan C., Hu P., Wu X., Yang W., Zhu Z. (2020). miR-199a-5p Exacerbated Intestinal Barrier Dysfunction through Inhibiting Surfactant Protein D and Activating NF-κB Pathway in Sepsis. Mediat. Inflamm..

[44] Pimenta R., Viana N.I., Dos Santos G.A., Candido P., Guimarães V.R., Romão P., Silva I.A., de Camargo J.A., Hatanaka D.M., Queiroz P.G.S. (2021). MiR-200c-3p Expression may be Associated with Worsening of the Clinical Course of Patients with COVID-19. Mol. Biol. Res. Commun..

[45] Mitchell M.I., Ben-Dov I.Z., Liu C., Ye K., Chow K., Kramer Y., Gangadharan A., Park S., Fitzgerald S., Ramnauth A. (2021). Extracellular Vesicle Capture by AnTibody of CHoice and Enzymatic Release (EV-CATCHER): A Customizable Purification Assay Designed for Small-RNA Biomarker Identification and Evaluation of Circulating Small-EVs. J. Extracell. Vesicles.

[46] Sodagar H., Alipour S., Hasani S., Aziz S.G.-G., Ansari M.H.K., Asghari R. (2022). The Role of microRNAs in COVID-19 with a Focus on miR-200c. J. Circ. Biomark..

[47] Yang Z., Hu Q., Huang F., Xiong S., Sun Y. (2021). The Prognostic Value of the SOFA Score in Patients with COVID-19. Medicine.

[48] Roepke R.M.L., Janzantti H.B.L., Cantamessa M.B., Machado L.F., Luckemeyer G.D., Gandolfi J.V., Besen B.A.M.P., Lobo S.M., Roepke R.M.L., Janzantti H.B.L. (2025). Predictive Performance of SAPS-3, SOFA Score, and Procalcitonin for Hospital Mortality in COVID-19 Viral Sepsis: A Cohort Study. Life.

[49] Li H., Liu L., Zhang D., Xu J., Dai H., Tang N., Su X., Cao B. (2020). SARS-CoV-2 and Viral Sepsis: Observations and Hypotheses. Lancet.

[50] Chen L., Zhang X., Shi P. (2025). Recent Advances in Biomarkers for Detection and Diagnosis of Sepsis and Organ Dysfunction: A Comprehensive Review. Eur. J. Med. Res..

[51] Ma L., Zhang Y., Hu F. (2020). miR-28-5p Inhibits the Migration of Breast Cancer by Regulating WSB2. Int. J. Mol. Med..

[52] Wang C., Wu C., Yang Q., Ding M., Zhong J., Zhang C.-Y., Ge J., Wang J., Zhang C. (2016). miR-28-5p Acts as a Tumor Suppressor in Renal Cell Carcinoma for Multiple Antitumor Effects by Targeting RAP1B. Oncotarget.

[53] Abdelaleem O.O., Mohammed S.R., El Sayed H.S., Hussein S.K., Ali D.Y., Abdelwahed M.Y., Gaber S.N., Hemeda N.F., El-Hmid R.G.A. (2022). Serum miR-34a-5p and miR-199a-3p as New Biomarkers of Neonatal Sepsis. PLoS ONE.

[54] Yu J., Chen J., Yang H., Chen S., Wang Z. (2019). Overexpression of miR-200a-3p Promoted Inflammation in Sepsis-induced Brain Injury through ROS-induced NLRP3. Int. J. Mol. Med..

[55] Welsh J.A., Goberdhan D.C.I., O’Driscoll L., Buzas E.I., Blenkiron C., Bussolati B., Cai H., Di Vizio D., Driedonks T.A.P., Erdbrügger U. (2024). Minimal Information for Studies of Extracellular Vesicles (MISEV2023): From Basic to Advanced Approaches. J. Extracell. Vesicles.

