# Supplementary material for: Extracellular Vesicle-Derived MicroRNAs’ Value in Diagnosing and Predicting Clinical Outcomes in Patients with COVID-19 and Bacterial Sepsis
Source: Int J Mol Sci. 2026 Jan 29;27(3):1334. doi: 10.3390/ijms27031334 (PMC12898072; doi:10.3390/ijms27031334)
Supplement: Supplementary file 1 [file ijms-27-01334-s001.zip › Table S1.pdf]

Table S1. Demographic and clinical characteristics of COVID-19 patients.

**Characteristics**	**Good prognosis COVID-19 Patients** (n=8)	**Poor prognosis COVID-19 Patients** (n=10)	***p*-value**
**Age, year, mean (± SD)**	76 (± 12.25)	61.80 (± 9.16)	**0.012**
**Gender ratio (M/F)**	5/3	6/4	>0.999
**Comorbidities**			
Diabetes Mellitus, n (%)	4 (50%)	5 (50%)	>0.999
Hypertension, n (%)	3 (37.5%)	4 (40%)	0.914
Obesity	1 (12.5%)	4 (40%)	0.195
Cancer	0	0	-
Smoking	0	2 (20%)	0.179
**MAP**	116.8 (± 14.01)	120.5 (± 21.52)	0.678
**In-hospital death, n (%)**	0	0	-
**SOFA score**	2 (2-2)	2.30 (± 1.34)	0.569
**Laboratory findings**			
White Blood Cell (10^9/L)	6.62 (± 2.35)	6.33 (4.45-9.97)	0.897
Hemoglobin (g/dL)	12.74 (± 1.49)	14.12 (± 1.26)	**0.049**
Platelets (10^9/L)	187.5 (158.5-325.8)	199.9 (± 69.93)	0.762
Creatinine (mg/dL)	0.98 (± 0.29)	0.93 (±0.25)	0.723
Bilirubin (mg/dL)	0.65 (± 0.20)	0.57 (± 0.20)	0.405
Lactate (mmol/L)	0.99 (± 0.22)	1.25 (± 0.39)	0.108
C Reactive Protein (mg/l)	53.86 (± 34.94)	102.3 (31.98-154.1)	0.171
Procalcitonin (ng/ml)	0.07 (0.06-0.38)	0.10 (± 0.05)	0.943

Mean (± SD) or median (IQR) as appropriate.
