# Supplementary material for: Extracellular Vesicle-Derived MicroRNAs’ Value in Diagnosing and Predicting Clinical Outcomes in Patients with COVID-19 and Bacterial Sepsis
Source: Int J Mol Sci. 2026 Jan 29;27(3):1334. doi: 10.3390/ijms27031334 (PMC12898072; doi:10.3390/ijms27031334)
Supplement: Supplementary file 1 [file ijms-27-01334-s001.zip › Table S2.pdf]

Table S2. Demographic and clinical characteristics of patients stratified for SOFA score.

Characteristics	SOFA 2-5 (n=17)	SOFA 6-10 (n=16)	*p*-value
**Age, year, mean (± SD)**	80 (73-88)	70.69 (± 12.38)	0.085
**Gender ratio (M/F)**	9/8	8/8	0.866
**COMORBIDITIES**			
Diabetes Mellitus, n (%)	4 (23.5%)	5 (31.2%)	0.498
Hypertension, n (%)	8 (47%)	12 (75%)	0.100
Obesity	2 (11.8%)	3 (18.8%)	0.576
Cancer	2 (11.8%)	5 (31.2%)	0.171
Smoking	2 (11.8%)	3 (18.7%)	0.935
**MAP**	97.67 (± 13.10)	82.20 (± 13.86)	**0.014**
**In-hospital death, n (%)**	1 (5.9%)	6 (37.5%)	**0.026**
**SOFA score**	4 (3-4.5)	8 (6-8.75)	**<0.0001**
**Primary Site of Infections**			
Pneumonia (Respiratory)	11 (64.7%)	7 (43.7%)	0.226
Abdominal (Gastrointestinal)	2 (11.8%)	4 (25.0%)	0.324
Urinary tract	2 (11.7%)	2 (12.5%)	0.948
Skin	3 (17.6%)	2 (12.5%)	0.680
**Laboratory Findings**			
White Blood Cell (10^9/L)	8.31 (± 3.45)	13.58 (9.08-20.80)	**0.007**
Hemoglobin (g/dL)	12.07 (±3.40)	12.40 (10.35-13.98)	0.824
Platelets (10^9/L)	150 (126-272)	234.5 (135.5-464.5)	0.214
Creatinine (mg/dL)	1.29 (0.85-1.87)	2.20 (1.01-2.80)	0.109
Bilirubin (mg/dL)	0.85 (± 0.47)	2.00 (0.75-6.00)	**0.006**
Lactate (mmol/L)	1.80 (1.00-6.37)	3.00 (1.25-4.80)	0.874
C Reactive Protein (mg/l)	86.80 (23.70-112)	101.6 (36.30-269.4)	0.338
Procalcitonin (ng/ml)	0.63 (0.13-6.41)	1.07 (0.14-8.41)	0.889

Mean (± SD) or median (IQR) as appropriate.
