# Supplementary material for: Extracellular Vesicle-Derived MicroRNAs’ Value in Diagnosing and Predicting Clinical Outcomes in Patients with COVID-19 and Bacterial Sepsis
Source: Int J Mol Sci. 2026 Jan 29;27(3):1334. doi: 10.3390/ijms27031334 (PMC12898072; doi:10.3390/ijms27031334)
Supplement: Supplementary file 1 [file ijms-27-01334-s001.zip › Table S3.pdf]

Table S3. Demographic and clinical characteristics of septic patients stratified for outcome during hospitalization.

Characteristics	Alive (n=26)	Dead (n=7)	*p*-value
**Age, year, mean (± SD)**	74.27 (± 12.16)	75.29 (±16.80)	0.858
**Gender ratio (M/F)**	14/12	3/4	0.606
**Comorbidities**			
Diabetes Mellitus, n (%)	6 (23.08%)	3 (42.9%)	0.297
Hypertension, n (%)	16 (61.5%)	4 (57.1%)	0.211
Obesity	3 (11.5%)	2 (28.6%)	0.265
Cancer	5 (19.2%)	2 (28.6%)	0.592
Smoking	3 (11.5%)	2 (28.6%)	0.265
**MAP**	94.18 (±1 3.80)	78.60 (± 15.31)	**0.042**
**SOFA score**	5.15 (± 2.41)	7.00 (± 1.73)	0.068
**Primary Site of Infections**			
Pneumonia (Respiratory)	14 (53.8%)	4 (57.1%)	0.876
Abdominal (Gastrointestinal)	6 (23.1%)	0	0.160
Urinary tract	2 (7.7%)	2 (28.6%)	0.133
Skin	5 (19.2%)	1 (14.3%)	0.763
**Laboratory Findings**			
White Blood Cell (10^9/L)	9.87 (6.47-13.00)	14.30 (± 7.43)	0.288
Hemoglobin (g/dL)	11.30 (9.90-13.93)	13.23 (± 2.72)	0.254
Platelets (10^9/L)	150 (119.5-294)	288 (229-600)	**0.042**
Creatinine (mg/dL)	1.47 (1.00 – 2.67)	1.90 (± 0.89)	0.682
Bilirubin (mg/dL)	0.95 (0.50 – 1.62)	2.80 (1.10 – 13.00)	0.076
Lactate (mmol/L)	1.60 (1.00-4.70)	5.12 (± 3.36)	0.150
C Reactive Protein (mg/l)	94.50 (38.70-202)	66.75 (± 60.30)	0.247
Procalcitonin (ng/mL)	0.47 (0.20-9.26)	1.07 (0.11-3.54)	0.747

Mean (± SD) or median (IQR) as appropriate.
