# Supplementary material for: Extracellular Vesicle-Derived MicroRNAs’ Value in Diagnosing and Predicting Clinical Outcomes in Patients with COVID-19 and Bacterial Sepsis
Source: Int J Mol Sci. 2026 Jan 29;27(3):1334. doi: 10.3390/ijms27031334 (PMC12898072; doi:10.3390/ijms27031334)
Supplement: Supplementary file 1 [file ijms-27-01334-s001.zip › Figure S1.pdf]

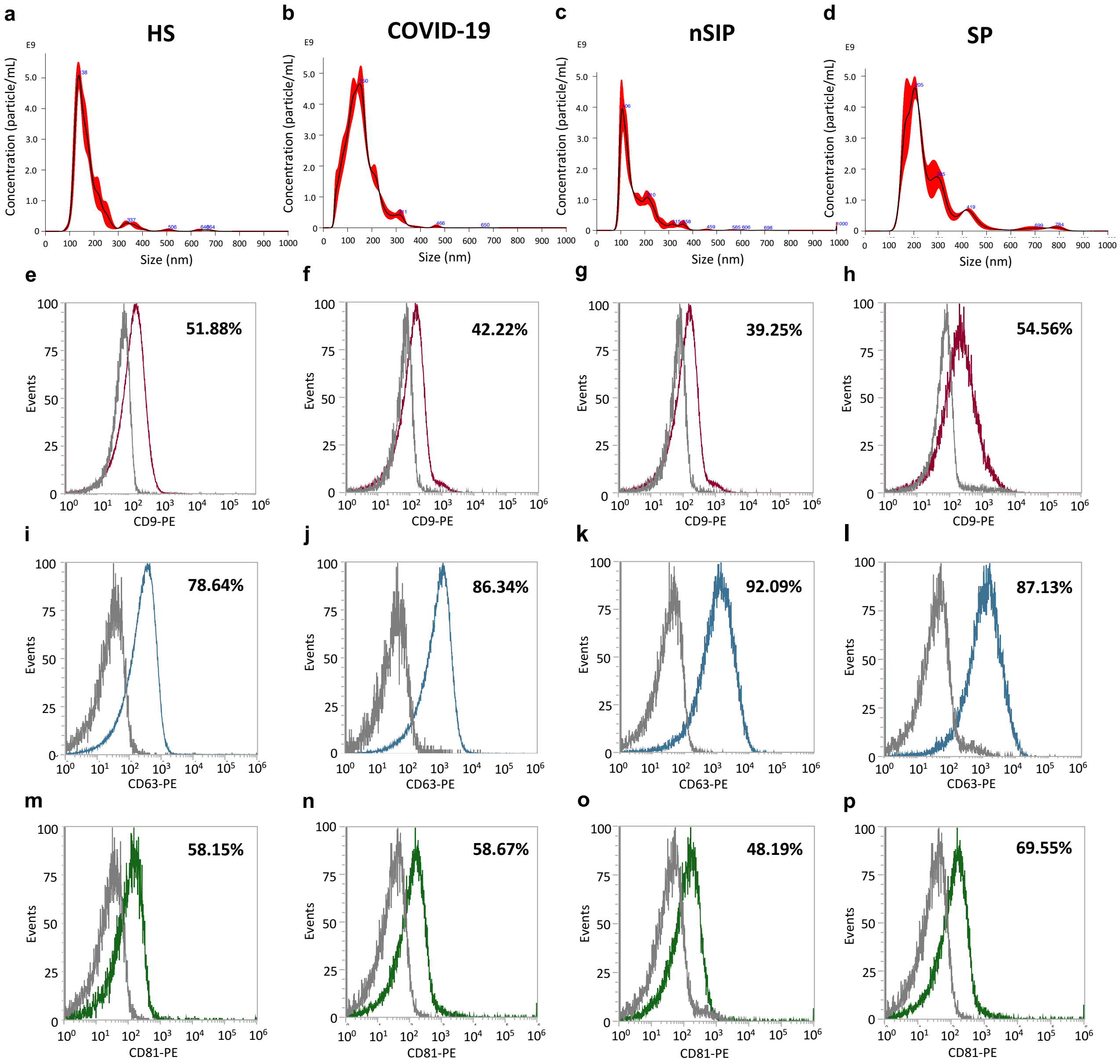


**Figure S1.** Representative images of Nanosight analysis referred to (**a**) healthy subjects (HS), (**b**) COVID-19 patients (COVID-19), (**c**) non-septic infected patients (nSIP), (**d**) septic patients (SP). Representative images of CD9 (red), CD63 (blu) and CD81 (green) by flow cytometry in HS (**e, i, m**), COVID-19 (**f, j, n**), nSIP (**g, k, o**), SP (**h, l, p**).
